# Where is the limit of prostate cancer biomarker research? Systematic investigation of potential prognostic and diagnostic biomarkers

**DOI:** 10.1186/s12894-019-0479-z

**Published:** 2019-06-06

**Authors:** Anika Kremer, Tobias Kremer, Glen Kristiansen, Yuri Tolkach

**Affiliations:** 1 Institute of Pathology, University Hospital of Bonn, Bonn, Germany; 2 Institute of Computer Science, Johannes Gutenberg-University Mainz, Mainz, Germany

**Keywords:** Prostate cancer, Biomarkers, Bioinformatics, mRNA expression, Prognostic, Diagnostic

## Abstract

**Background:**

The identification of appropriate biomarkers is essential to support important clinical decisions in patients with prostate cancer. The aim of our study was a systematic bioinformatical analysis of the mRNA expression of all genes available for the prostate adenocarcinoma cohort of The Cancer Genome Atlas (TCGA), regarding their potential prognostic and diagnostic role.

**Methods:**

The study cohort comprises 499 patients (TCGA prostate cancer cohort). mRNA expression data were available for approx. 20,000 genes. The bioinformatical statistical pipeline addressed gene expression differences in tumor vs. benign prostate tissue (including gene set enrichment analysis, GSEA) in samples from tumors with different aggressivenesses (Gleason score), as well as prognostic values in multistep survival analyses.

**Results:**

Among all genes analyzed, 1754 were significantly downregulated and 1553 genes were significantly upregulated in tumor tissue. In GSEA, 16 of 30 top enriched biological processes were alterations of epigenetic regulation at different levels. Significant correlation with Gleason Score was evident for 8724 genes (range of Pearson r-values 0.09–0.43; all *p* < 0.05). In univariate Cox regression analyses, mRNA expression of 3571 genes showed statistically significant association with biochemical recurrence-free survival with a range of hazard ratios 0.3–3.8 (*p*-value 7.4e− 07 to 0.05). Among these, 571 genes were independently associated with biochemical recurrence in multivariate analysis. Access to the full database including results is provided as supplement.

**Conclusions:**

In our systematic analysis we found a big number of genes of potential diagnostic and prognostic value, many of which have not been studied in prostate cancer to date. Due to the comprehensive nature of this analysis and free access to the results, this study represents a reference database for prostate cancer researchers which can be used as a powerful tool for validation purposes and planning of new studies.

**Electronic supplementary material:**

The online version of this article (10.1186/s12894-019-0479-z) contains supplementary material, which is available to authorized users.

## Background

Prostate cancer (PCa) is one of the most common cancers in men worldwide [1]. Once the prostate carcinoma is diagnosed, it is considered well treatable if recognized at an early stage. Though, over the past years, PCa therapy came into the focus of criticism due to its potential for overtreatment by e.g. radical prostatectomy [2, 3]. However, rendering reliable diagnoses and prognoses of the progression of the disease based on tumor grading and modern classifications is impeded by its high degree of morphological and molecular genetic heterogeneity [4, 5]. Therefore, its high rate of occurrence and frequent heterogeneity make PCa not only a crucial but also a complex and challenging research target in clinical and research settings.

The identification of appropriate biomarkers is therefore essential to drive important clinical decisions in patients with prostate cancer [6]. Hundreds of studies focusing on new biomarkers are being published every year for more than 40 years now [7]. Three main biomarker branches have been established: diagnostic markers, that identify patients at risk of prostate cancer using serum/ urine as substrate, or diagnostic immunohistochemistry during biopsy evaluation, prognostic markers, which give an idea of a certain clinical outcome, e.g. biochemical recurrence after radical treatment and predictive markers, predicting a response to specific, usually medicament-based therapy.

Prognostic biomarkers, even after their almost 40-year-long research way, are still not recommended for utilization in clinical routine although several of them (in form of commercial gene expression signatures) are considered as potential candidates. However, with no data currently available about their clinical relevance at long term, also prospective validation is lacking [8].

Given that after 40 years of research multiple studies appear constantly in the literature addressing single genes or their combinations in the prognostic setting, the aim of our study was to define the limits of biomarker research in patients with prostate cancer, primarily through the identification of potential targets not yet investigated. This comprehensive, holistic approach cannot only serve as an outline of the immense possibilities that are still left for prognostic and diagnostic biomarker research but could also be used as a starting point for important discussions such as the prioritization of research targets and changes in research methodology for future prostate cancer research.

## Methods

### Patient cohort

**Fig. 1:**
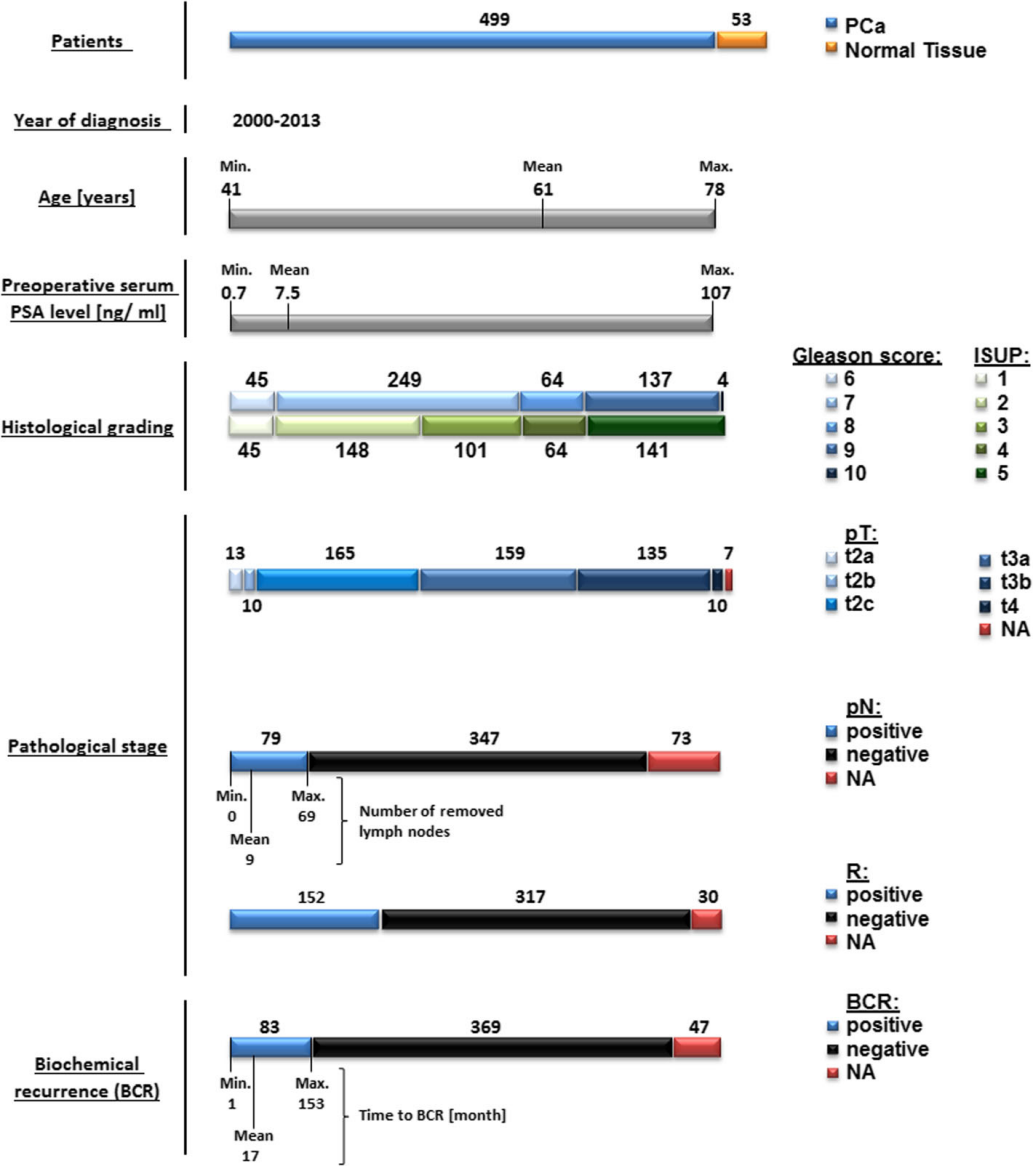
Clinicopathological characteristics of patients

This study comprised a total of 499 patients from the prostate adenocarcinoma cohort of The Cancer Genome Atlas (TCGA). Mean age of the patients was 61.0 years (range 41–78 years). Clinicopathological information was available for all patients (Fig. 1). Follow-up information with biochemical recurrence as an endpoint was available for 452 patients, 83 of which have developed a biochemical recurrence. Median follow-up time was 16.9 months (range 1–153 months).

### Quality control of clinical data

The raw clinical data (version 28.01.2016) was pre-processed and organized in a database as follows: 1) selection of relevant clinicopathological parameters (age, serum PSA level, pathological staging and grading, follow-up information); 2) All parameters were controlled for consistency, duplicates were removed.

### mRNA expression data

mRNA expression data were generated using the Illumina HiSeq 2000 RNA Sequencing platform (Version 2; data version 28.01.2016). RNA expression was available for tumor samples of all patients and for additional 53 samples with normal tissue normalize according to TCGA protocol. Using barcode as an identifier, tumor and normal tissue samples were extracted separately with further merging of RNA expression data for tumor samples to clinical data. After excluding the genes with duplicate names and missing expression, mRNA expression of 20,500 genes was available for analysis. From this list, further 2819 genes were excluded due to absent or very low mRNA expression values (median = 0 reads), leaving 17,681 genes in the final analysis.

### Bioinformatical approach / statistics

All statistical analyses were performed using R (R Foundation for Statistical Computing; version 3.5.0). The packages used were pastecs, TCGAbiolinks, limma, edgeR, KMsurv, survMisc, rms, stringi, Hmisc, tidyverse and doParallel.

**Fig. 2:**
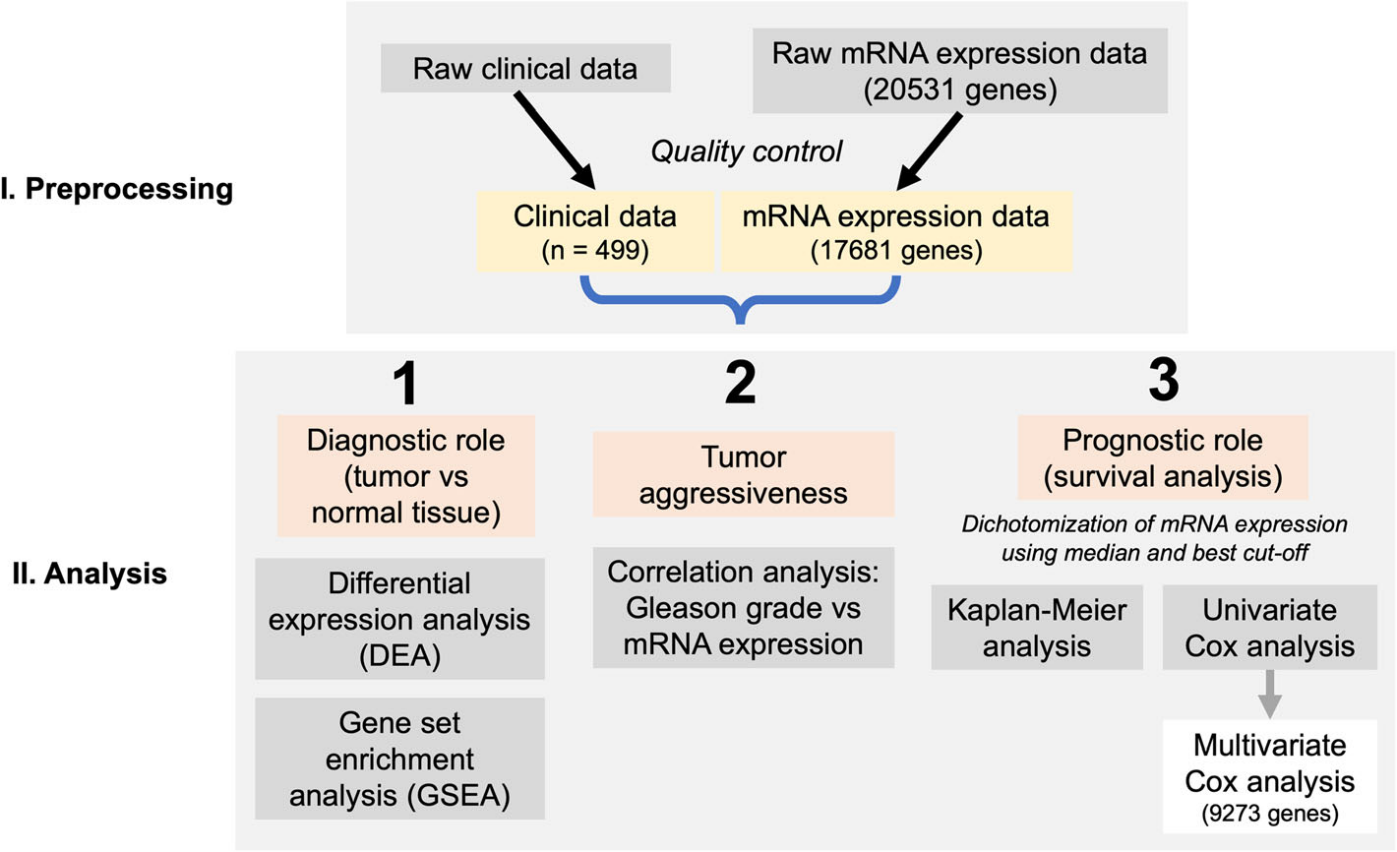
Bioinformatical pipeline for the analysis of the prognostic role of gene expression

The fully automatized bioinformatical pipeline is outlined in Fig. 2. In brief, for survival analyses, dichotomization of mRNA expression was carried out using 1) median level of mRNA expression and 2) cut-off optimization (best cut-off). The best cut-off was selected using the survMisc package (automatized systematic univariate Cox regression-based analysis of all available cut-offs for mRNA expression of single genes). Survival analyses were conducted using univariate and multivariate Cox proportional hazards regression. Kaplan-Meier estimates were calculated using both the best cut-off and median for each gene with accompanying log-rank test and automatic generation of Kaplan-Meier curves for all genes. The inclusion criterion for multivariate analysis was a *p*-value < 0.05 in univariate analysis.

Correlation analyses were performed to identify the associations of clinical variables (Gleason Score) with the mRNA expression of single genes (Pearson correlation coefficient (r) and p-level). Pairwise comparison of the gene expression of normal and tumor tissue was carried out using a negative binomial generalized log-linear model and correction for false discovery rate (FDR).

### Gene set enrichment analysis (GSEA)

GSEA for tumor versus normal tissue was performed using the GSEAPreranked tool in javaGSEA Application (The Broad Institute, Inc., Massachusetts Institute of Technology and Regents of the University of California). Single genes were ranked based on the logFC parameter stemming from differential gene expression analysis. Gene sets from the Hallmark collection (well-defined biological states or processes, *n* = 50) and the Gene Ontology database (GO biological processes, *n* = 4436) were used for GSEA as provided by Molecular Signatures Database (MSigDB) v6.2 (The Broad Institute, Inc., Massachusetts Institute of Technology and Regents of the University of California). GSEA was performed using the following setup: number of permutations – 1000, enrichment statistic – weighted, gene set size restriction – 15-500 genes, FDR cut-off – 0.25.

## Results

### Tumor vs normal tissue

Among all genes analyzed, 1754 were significantly downregulated in tumor tissue (fold change (FC) > 2, logFC < − 1.0; FDR range from 0.05 to 3.3e-275). 133 of these genes showed profound downregulation with logFC < − 3.0 (maximal logFC -9.7; FDR 1.4e− 09 – 3.3e-275).

Another 1553 genes were significantly upregulated in tumor tissue (FC > 2, logFC > 1; FDR range from 0.047 to 1.6e-51) with very high levels of upregulation (logFC > 3; maximal logFC 9.9) in 123 genes (FDR 0.02–4.1e-36). For full information see Additional file 2: Table S1.

**Fig. 3:**
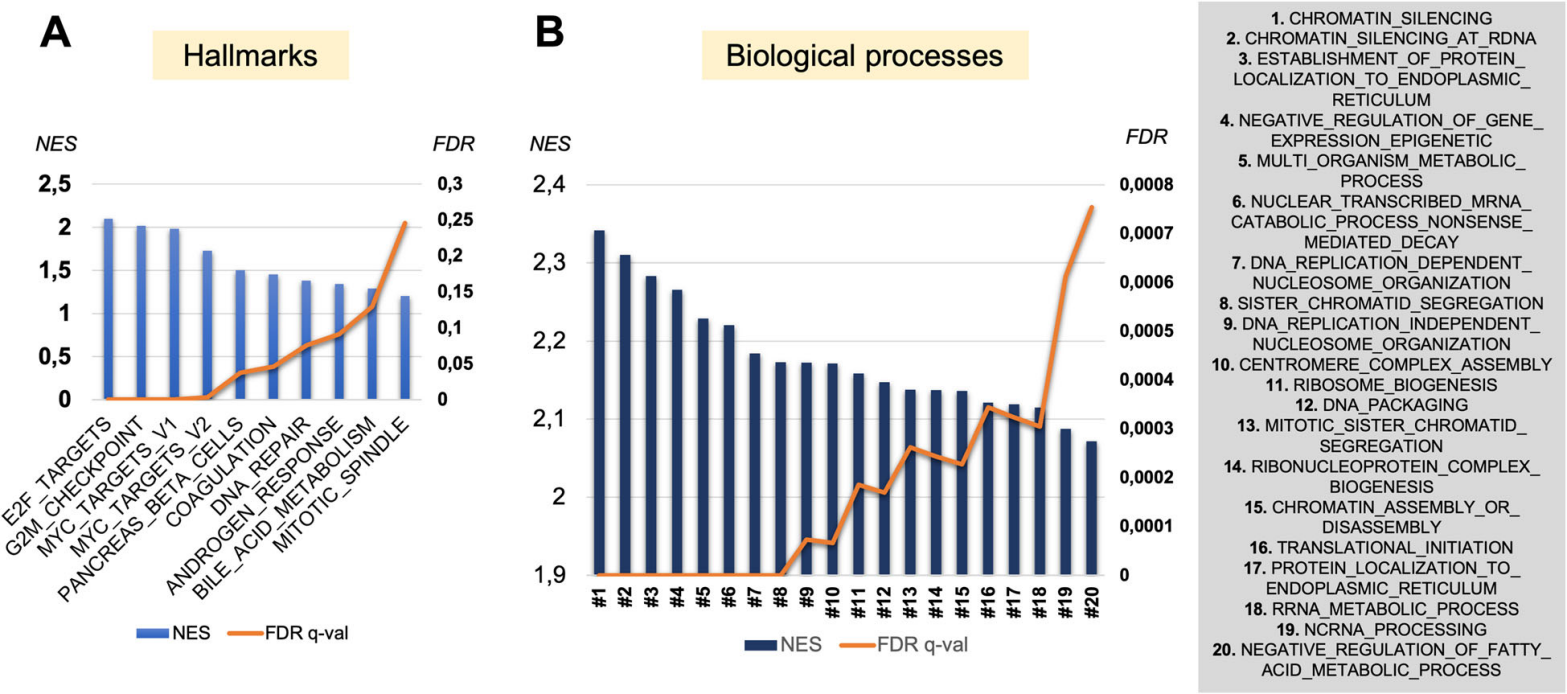
The results of Gene Set Enrichment Analysis (GSEA) in tumor tissue compared to normal tissue. Two collections of gene sets from Molecular Signatures Database (MSigDB) were analyzed: **a**) Hallmarks of well-defined biological states of processes (10/10 enriched signatures for tumor tissue are shown). **b** Gene Ontology database: biological processes (top 20/809 enriched signatures are shown). False discovery rate was set at a cut-off of 0.25; 1000 permutations were made for every analysis. Abbreviations: NES – normalized enrichment score (main metrics of GSEA); FDR – false discovery rate

In GSEA analysis, 10 Hallmark gene sets and 809 gene sets from the GO Biological Processes collection were enriched in tumor tissue with FDR < 0.25 (Fig. 3, Additional file 3: Table S2). Multiple Biological Processes enriched in tumor tissue were related to altered epigenetic regulation (chromatin organization, gene silencing).

### Correlation with Gleason score (tumor aggressiveness)

Of 17,681 genes, 8724 genes showed significant correlation levels to the International Society of Urological Pathology (ISUP) histological grading group of the tumor (based on the Gleason Score) with *p*-values ranging from 10e-24 up to 0.05 and range of Pearson r-values of 0.09–0.43. From this list, 5557 genes were positively correlated to ISUP grouping, while 3167 genes were negatively correlated. Top 20 genes with the highest levels of positive and negative correlation are presented in Table 1 (for full analysis see Additional file 4: Table S3; for correlation analysis high grade (≥4 + 4) vs low grade tumors see Additional file 5: Table S4).

**Table 1:** Top 20 genes with the highest levels of mRNA expression correlation to ISUP grading group of the tumor

Positive correlation			Negative correlation		
Gene	ISUP / Pearson r	ISUP / p-value	Gene	ISUP / Pearson r	ISUP / *p*-value
TROAP	0,408	2,21e-21	SH3RF2	−0,429	9,99e-2
CBX1	0,408	2,55e-21	RNF185	−0,405	4,85e-21
ABCC5	0,404	6,19e-21	VPS36	−0,400	1,74e-20
DONSON	0,403	8,80e-21	ACP2	−0,397	3,60e-20
SMC4	0,400	1,55e-20	GEMIN4	−0,388	2,70e-19
KIF20A	0,400	1,65e-20	KIAA0319L	−0,387	3,03e-19
SPAG5	0,399	2,29e-20	KCNK6	-0,384	6,28e-19
NCAPG2	0,396	4,57e-20	DPP4	-0,383	7,49e-19
KIF23	0,395	5,42e-20	CCDC149	-0,381	1,33e-18
FAM72D	0,394	6,57e-20	EPHX2	-0,380	1,65e-18

### Prognostic role of mRNA expression (survival analyses)

#### Univariate cox regression

**Fig. 4:**
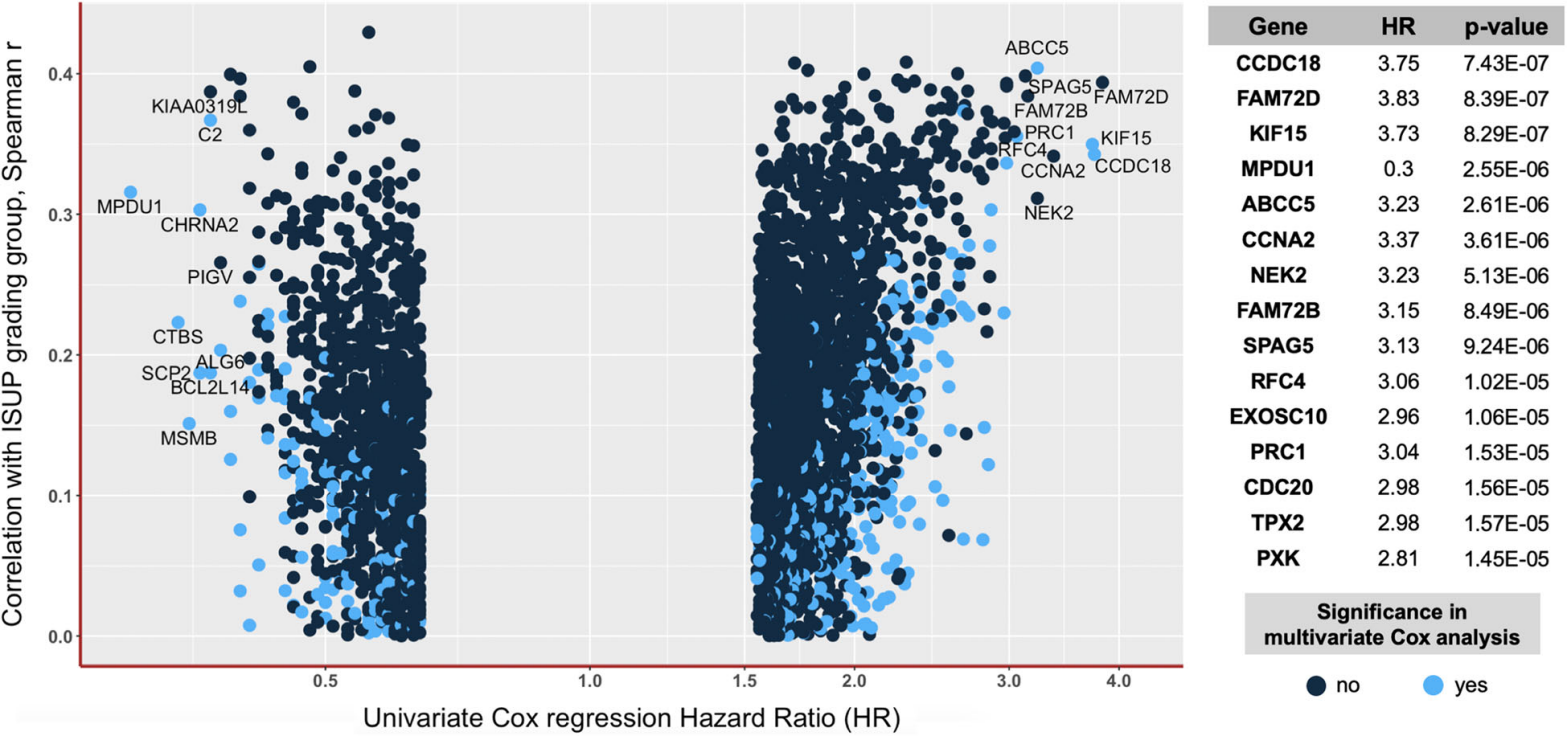
All genes with statistically significant association of mRNA expression with biochemical recurrence in univariate Cox regression analysis (all *p* < 0.05, median of expression as dichotomization cut-off) stratified according to univariate Hazard Ratio (HR) and correlation level between ISUP grad group of the tumor and mRNA-expression of the gene. Blue spots represent the genes which are independently associated with biochemical recurrence-free survival in multivariate Cox regression analysis. Top 15 genes with highest levels of statistical significance in univariate Cox analysis are highlighted with detailed outputs from univariate Cox regression analysis

In univariate Cox regression analyses (Fig. 4), 3571 of 17,681 genes showed a statistically significant association with biochemical recurrence (BCR)-free survival of patients with a range of hazard ratios (HR) 0.3–3.8 (*p*-values 7.4e− 07 to 0.05), when dichotomized using median of expression (additional 5719 genes demonstrated statistical significance with *p* < 0.05 using best cut-off for dichotomization). Of 3571 genes significantly associated with BCR (dichotomization using median), 827 were not significantly correlated with the ISUP grading group of the tumor. Higher mRNA expression was prognostically unfavorable for 2390 genes and favorable for 1181 genes (top 20 genes outlined in Fig. 4). Full information about the prognostic significance of mRNA expression of single genes in univariate analysis using median and best cut-off for dichotomization is available as Additional file 4: Table S3.

#### Multivariate cox regression

Pathological staging of the tumor (pT: pooled pT3/4 vs pT2), ISUP histological grade group of the tumor, presence of lymph nodes metastases (pN1 vs pN0) and status of the resection margins (R1 vs R0) were included into multivariate Cox regression models, together with the expression of single genes which showed statistically significant association with BCR-free survival in univariate Cox regression analysis (3571 genes with dichotomization using median of expression and 9273 genes with dichotomization using the optimized cut-off).

**Fig. 5:**
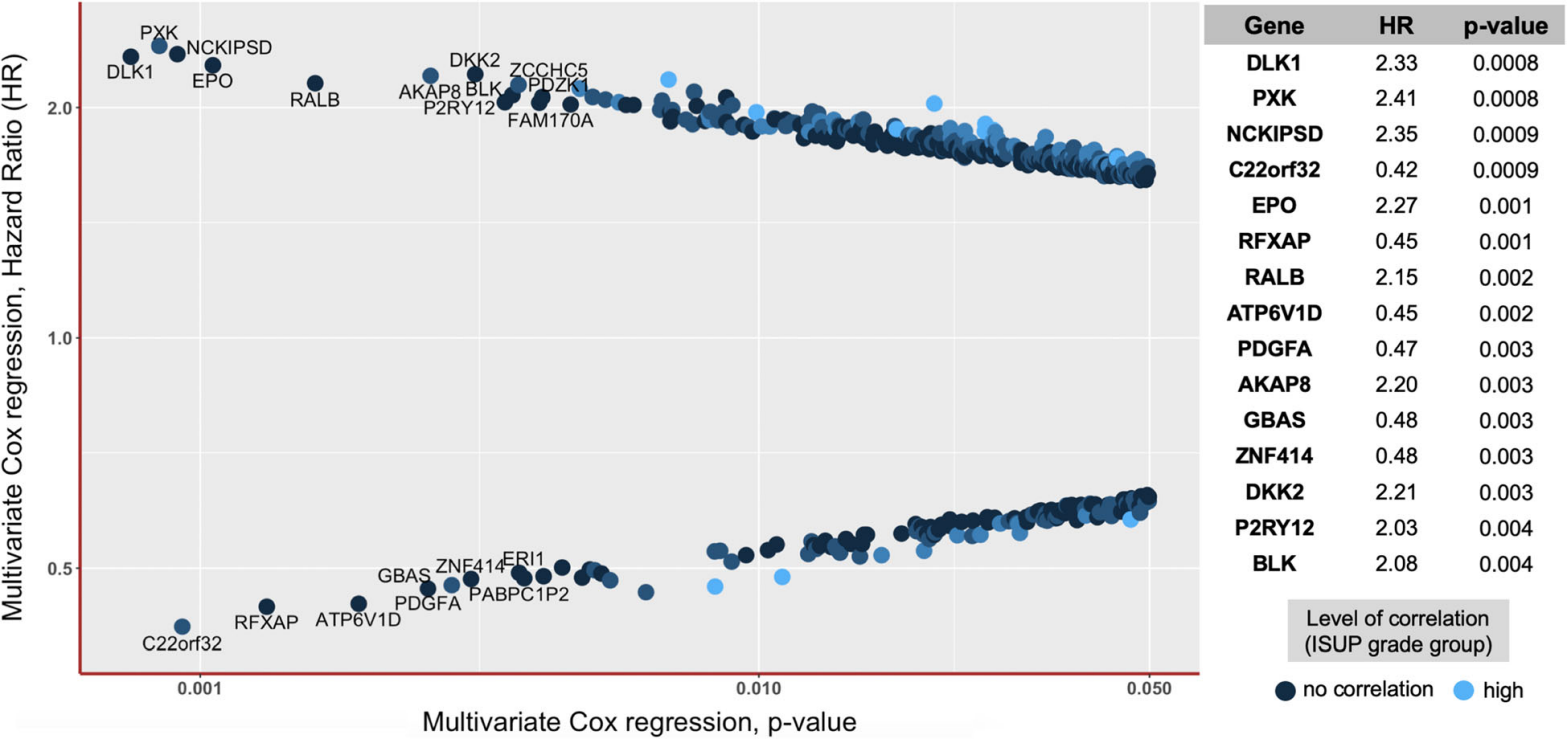
All genes with statistically significant association of mRNA expression with biochemical recurrence in multivariate Cox regression analysis (all p < 0.05, median of expression as dichotomization cut-off) stratified according to multivariate Hazard Ratio (HR) and multivariate analysis p-level. Colors represent correlation levels to the ISUP grade group of the tumor (from dark blue = Spearman r < 0.1 and no significant correlation up to light blue = Spearman r > 0.3, *p* > 0.05). Detailed outputs for top 15 genes from multivariate Cox regression analysis are provided on the right side

**Fig. 6:**
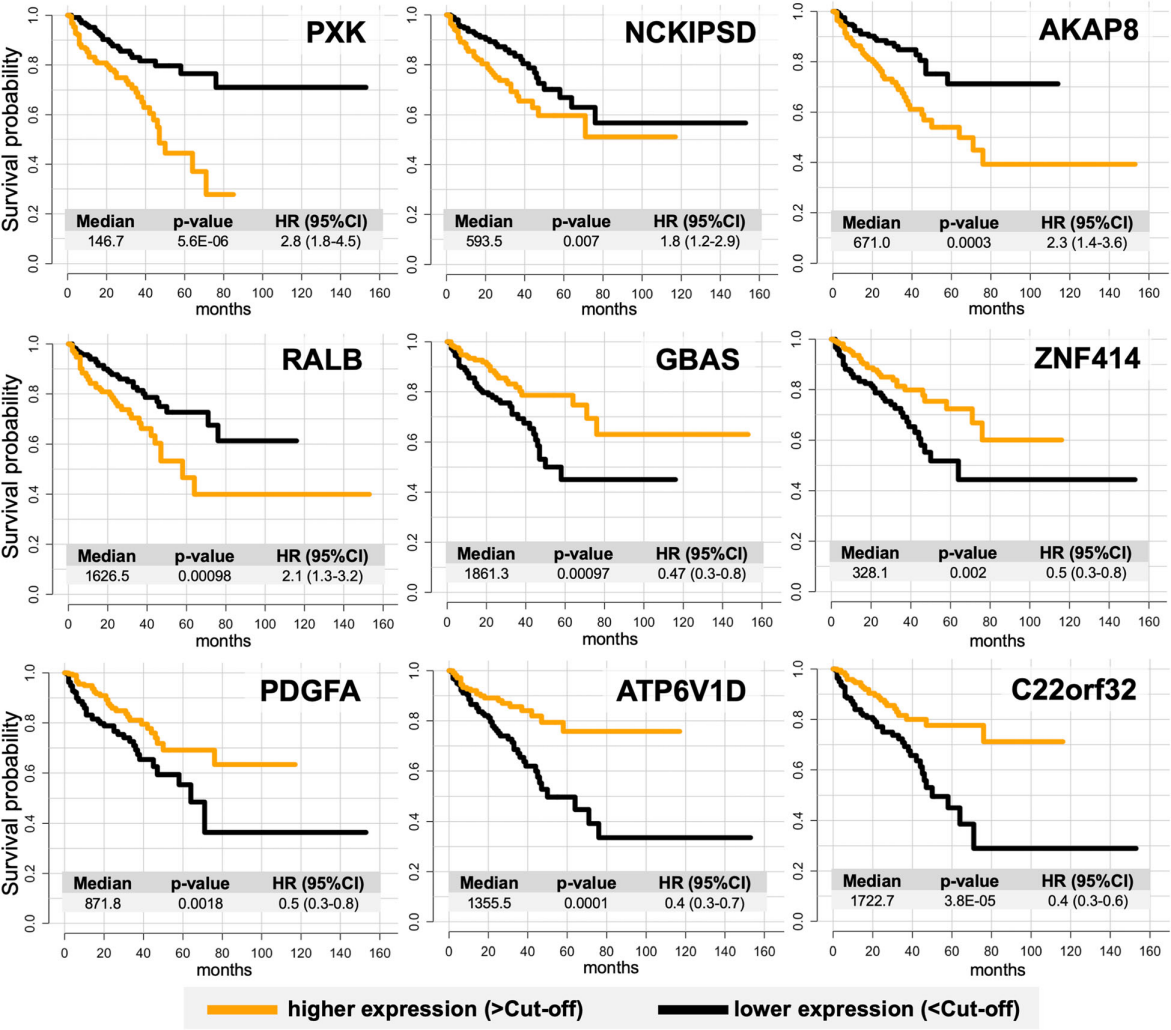
Kaplan-Meier curves for nine top-ranked genes with substantial expression in prostate cancer, statistically significant and independently associated with biochemical recurrence-free survival of the patients with prostate cancer in multivariate Cox regression analysis (median of expression as dichotomization cut-off). Abbreviations: Median – median of mRNA expression, HR – hazard ratio, l95% CI and u95% CI – lower and upper 95% percentile of the confidence interval (CI), low expression – expression levels below cut-off, high expression – expression levels above cut-off

Among 3571 (median as cut-off) / 9273 (best cut-off) included genes, 571 / 2435 genes, respectively, showed statistically significant association with biochemical recurrence (Fig. 5). With median as cut-off, multivariate Cox regression *p*-values for single genes ranged from 0.0005 to 0.05 and hazard ratios from 0.40 to 2.47 (best cut-off p-value range 2.1e− 05 – 0.05, HR range 0.10–12.31). Kaplan-Meier curves for genes most significantly and independently associated with BCR-free survival in multivariate analysis are presented in Fig. 6. Full information about the prognostic significance of mRNA expression of all single genes in the multivariate analysis with dichotomization using median and best cut-off is available as additional file 6: Table S5.

Of 571 genes independently associated with BCR-free survival (median as cut-off), mRNA expression of 276 genes was significantly correlated with the ISUP grade group of the tumor (Pearson r > 0.10, *p* < 0.05).

Characterization of the top 50 genes independently associated with BCR-free survival with analysis of their potential for further investigations in patients with prostate cancer is presented in Additional file 7: Table S6.

## Discussion

The identification of appropriate biomarkers is essential to drive important clinical decisions in patients with prostate cancer [7]. Hundreds of studies focusing on new biomarkers are being published every year since the 1980th, addressing three main biomarker branches (diagnostic, prognostic, and predictive). Even though diagnostic markers have found their niche in the clinical practice (selection of patients at risk of prostate cancer for biopsy based on serum (e.g. prostate-specific antigen, four kallikrein score) or urine analysis (RNA expression of PCA3 [9], or of gene pair HOXC6 / DLX1 [10]) [8], immunohistochemistry during primary diagnosis or metastatic disesase [11], theranostic targets for imaging, such as prostate-specific antigen [12]), prognostic biomarkers are still not recommended by professional guidelines, despite 40 years of intensive research in patients with prostate cancer.

The aim of our study was to delineate the limits of prostate cancer prognostic and diagnostic biomarker research and to show the extent of perspective targets have not been studied in prostate cancer yet. For this purpose, we used a well-characterized primary hormone-naïve prostate cancer cohort from TCGA with 499 patients, representing 499 tumor and 53 normal tissue samples. The limits of diagnostic and prognostic biomarker research could be investigated with the use of this cohort as it includes data regarding the status of almost all genes. We have selected RNA expression data of all genes called using RNAseq approach as a surrogate for their functional relevance, and systematically approached the questions of the diagnostic and prognostic role of single genes using our automatized bioinformatical pipeline (Fig. 2).

As for the prognostic role of these genes during the statistical stages of Kaplan-Meier, univariate and multivariate Cox-regression analysis, we have cleared out 571 genes with independent prognostic significance for biochemical recurrence after radical prostatectomy using strict rules prescribed by REMARK criteria [13] (especially, using the median of expression as cut-off for dichotomization). Even more genes (overall 2435 genes) carried independent prognostic value under relaxed criteria (optimized cut-off for dichotomization). Although prone to statistical bias, using the best cut-off for dichotomization is logical from a biological point of view (e.g., only a small part (not the half) of carcinomas could have special aggressiveness features delineated by gene expression). Therefore, these genes also could and should be considered as potential candidates for further characterization.

Interestingly, when performing a detailed analysis of the top 50 genes showing independent prognostic value (Additional file 7: Table S6), 40 of them (80%), as for the actual state of published research, have not been studied in prostate cancer, yet. 31 of these genes were shown to be of some significance for other cancer types. This gives us a broad perspective of how much research effort and time we should invest to cover all potentially relevant targets which have not been in the scope of prostate cancer researchers yet.

As for the diagnostic role of these genes, we hereby provide a comprehensive analysis based on two approaches: 1) differential expression in tumor vs. normal tissue, and 2) correlation of mRNA expression with Gleason-Score / ISUP-grade group of the tumor. The first part of this analysis provides a big number of potential targets which are highly upregulated (*n* = 123) or downregulated (*n* = 133) in tumor tissue with fold change (FC) more than 8 (logFC > 3) with multiple genes showing less pronounced, however, still significant up- and downregulation.

Some of these genes were already extensively studied in prostate cancer, mostly those upregulated in tumor tissue: SPINK1 [14] (Top17, logFC 4.8), ETV4 [15] (Top63, logFC 3.6), PCA3 [9] (Top79, logFC 3.5), TDRD1 [16, 17] (Top86, logFC 3.4), AMACR [18] (Top105, logFC 3.2), DLX1 [10] (Top110, logFC 3.1), HOXC6 [10] (Top268, logFC 2.3). However, others are still representing potential diagnostic targets at different stages of clinical decision making (before diagnosis, after first negative biopsy, control of recurrence, immunohistochemical diagnosis of prostate cancer on biopsy, theranostic targets). Between the above mentioned highly upregulated genes and several genes used as targets in clinical practice such as FOLH1 coding PSMA (logFC 1.7, Top622 among all upregulated genes), there is a gap of approx. 500 genes which could represent potential diagnostic targets.

Interestingly, several well-known genes, despite being recommended for their utility in the identification of patients at risk of prostate cancer at RNA expression level, are by far not in the top of this list (e.g. in urine; PCA3, HOXC6, DLX1 [9, 10]). Hence, these results once again outline the need for further research on diagnostic biomarkers.

Gene set enrichment analysis (GSEA) for differentially expressed genes allowed us to detect many biological processes/pathways altered in tumor tissue. Interestingly, among the top 30 biological processes altered in prostate cancer, 16 (53.3%) were related to epigenetic mechanisms, such as chromatin functioning/ organization, and epigenetic gene silencing (Fig. 3, Additional file 3: Table S2). The epigenetics of prostate cancer are well studied at the level of DNA methylation [19], however, more studies investigating broader epigenetic mechanisms related to chromatin organization and functioning are warranted for a further comprehension of the prostate cancer biology.

In general, our study and associated supplementary materials including full results of the performed analyses represent are a very useful reference database for those researchers willing to validate the results of their studies involving different levels of gene expression (mRNA, methylation analysis, protein expression) in patients with prostate cancer.

The limitations of our study are mainly associated with the inherent limitations of the TCGA cohort and should be considered using this material as a reference point. Due to the relatively short follow-up period (median 17 months, range 1–153 months), the findings of our analyses are more useful for patients with higher Gleason scores, as they develop BCR earlier. Among 45 patients with Gleason Score 3 + 3 = 6, only 3 patients have developed BCR to the end of follow-up compared to 80 patients with BCR among patients with other Gleason scores. To address this point, we have carried out a multivariate Cox regression analysis separately for patients of all ISUP grade groups and for patients of ISUP grade groups ≥2 (Additional file 8: Table S7). In conclusion, the results showed minimal discrepancies in the resulting set of independent prognostic biomarkers and therefore, the robustness of our initial findings.

Furthermore, despite the high quality of the TCGA cohort, a validation cohort would be needed to verify the results, as it was shown earlier that a significant number of genes (mainly from small studies) do not pass the validation landmark [20]. Also, it is important to mention, that the patient cohort that was used in this study is a post-prostatectomy cohort of patients with primary hormone-naïve prostate cancer and the results, therefore, could be only in the restrictive manner extended to the patients with metastatic and castration-resistant prostate cancer.

Moreover, the analyses carried out involved only RNA expression levels of the genes. In the modern era of multi-omics and presence of multiple aspects of alternative regulation of gene function from transcription to protein function, this should be respectively interpreted. Only one tumor sample per patient was analyzed by TCGA which can introduce a bias related to undersampling of the tumor, given high levels of prostate cancer morphological and molecular genetic heterogeneity [5]. Accordingly, validation studies should address this important aspect of prostate cancer biology.

We believe that our study should also be a starting point for an important discussion. Although the particular advantage of our study is a comprehensive outline of the immense possibilities that are still left for prognostic and diagnostic biomarker research, it poses one important question (taking in account the almost 40-year-long portfolio of translational biomarker research which still didn’t find its way into clinical practice): what should be the priority of the future prostate cancer research and how do we need to change our research methodology to overcome the above mentioned issues of failing clinical relevance of biomarkers (especially prognostic) studied before? There is still no definitive answer to this question. However, it is more or less clear that single targets will probably only make sense in a diagnostic, but not in a prognostic setting. The optimal methodological approach for the development of prognostic biomarkers remains to be established and probably will account for pathway-oriented genetic and epigenetic changes at different levels (mutations, copy-number rearrangements, DNA methylation, chromatin remodeling, mRNA and ncRNA expression, posttranslational modifications, protein expression) and at different time points to account for tumor evolution and tumor heterogeneity [4, 21–23].

## Conclusions

Our study provides a comprehensive overview of the prognostic and diagnostic mRNA biomarkers in patients with primary prostate cancer, both already studied and, more importantly, not yet addressed in prostate cancer. Interestingly, several of them show a great potential for further research. These findings could be used as a reference point for further biomarker research and validation data for ongoing projects by most prostate cancer researchers. A full database including the results is provided as Supplement to be used as an everyday tool.

## Additional files

Additional file 1: Supplementary Data. List of abbreviations. (PDF 29 kb)

Additional file 2: **Table S1** Pairwise comparison of the gene expression of normal and tumor tissue using negative binomial generalized log-linear model and correction for false discovery rate (FDR). (TXT 2100 kb)

Additional file 3: **Table S2** Full results of Gene Set Enrichment Analysis (GSEA) for tumor vs normal tissue using Gene Ontology Biological Processes database. (TXT 131 kb)

Additional file 4: **Table S3** Correlation between ISUP histological grade group of the tumor (Grade groups 1–5) and mRNA expression of single genes, univariate Cox-regression analysis with biochemical recurrence as an endpoint. Full list of genes (*n* = 17,681). (TXT 3078 kb)

Additional file 5: **Table S4** Correlation between ISUP histological grade group of the tumor (dichotomized as high-grade, ≥4 + 4, vs low-grade tumors) and mRNA expression of single genes. Correlation analysis for ISUP grade groups (Groups 1–5) is provided in parallel for comparison. Delta of Pearson *r* correlation levels is calculated for these 2 different settings. (TXT 2104 kb)

Additional file 6: **Table S5** Full results of the multivariate Cox regression analysis for single genes. (TXT 1949 kb)

Additional file 7: **Table S6** Top 50 genes with statistically significant and independent association with biochemical recurrence-free survival in multivariate Cox regression analysis (dichotomization using median) in patients with prostate cancer. Estimates of their perspectivity for further research are provided. (PDF 65 kb)

Additional file 8: **Table S7** Results of the multivariate Cox regression analysis for single genes separately for patients of ISUP grade groups 2–5. (TXT 1940 kb)

## Data Availability

Clinical dataset and mRNA expression database was downloaded from the Broad GDAC Firehose homepage (gdac.broadinstitute.org). Comprehensive databases with stratified analyses generated during this study are included in this published article as Additional file 1: Supplementary data.

## Abbreviations

BCR: Biochemical recurrence
FC: Fold change
FDR: False discovery rate
ISUP: International Society of Urological Pathology
PCa: Prostate cancer
TCGA: The Cancer Genome Atlas
