# Supplementary material for: Where is the limit of prostate cancer biomarker research? Systematic investigation of potential prognostic and diagnostic biomarkers
Source: BMC Urol. 2019 Jun 6;19:46. doi: 10.1186/s12894-019-0479-z (PMC6554887; doi:10.1186/s12894-019-0479-z)
Supplement: Supplementary file 1 — Supplementary Data. List of abbreviations. (PDF 29 kb) [file 12894_2019_479_MOESM1_ESM.pdf]

Supplementary Data: List of abbreviations,

used in the Supplementary materials

Best/ Best_coff	Best cut-off
Cor_delta	Difference in correlation levels calculated for ISUP grade groups 1-5 vs dichotomized low-/high-grade tumors
Cor_isup_p_val	Correlation between ISUP grading group and p-Value
Cor_isup_r	Correlation between ISUP grading groups (1-5) and gene expression; Pearson correlation coefficient (r)
Cor_isup_hg_r	Correlation between high-grade/low-grade tumors and gene expression; Pearson correlation coefficient (r)
ES	Enrichment score (GSEA)
FDR	False discovery rate
GSEA	Gene Set Enrichment Analysis
GO	Gene ontology
HR	Hazard ratio
ISUP	International Society of Uropathology (ISUP grade groups)
isup_hg_lg_ttest_p_val	Comparisons of expression in low- vs high-grade tumors, t-test value
isup_hg_lg_wil_p_val	Comparisons of expression in low- vs high-grade tumors, Wilcox p value (Mann Whitney U test)
isup_lg_mean	Mean of expression in patients with low-grade tumors
isup_hg_mean	Mean of expression in patients with high-grade tumors
L95	Lower 95% quantile
LogCPM	Log counts per million
LogFC	Log fold-change
LR	Left–right asymmetry
Med	Median
Multi	Multivariate cox analysis
NES	Normalized enrichment score (GSEA)
Normal	Normal Tissue
P/ PValue	p-Value
Tumor	Tumor Tissue
U95	Upper 95% quantile
Uni	Univariate cox analysis
