# Supplementary material for: Where is the limit of prostate cancer biomarker research? Systematic investigation of potential prognostic and diagnostic biomarkers
Source: BMC Urol. 2019 Jun 6;19:46. doi: 10.1186/s12894-019-0479-z (PMC6554887; doi:10.1186/s12894-019-0479-z)
Supplement: Supplementary file 7 — Table S6 Top 50 genes with statistically significant and independent association with biochemical recurrence-free survival in multivariate Cox regression analysis (dichotomization using median) in patients with prostate cancer. Estimates of their perspectivity for further research are provided. (PDF 65 kb) [file 12894_2019_479_MOESM7_ESM.pdf]

**Suppl. Table VI:**

Top 50 genes with statistically significant and independent association with biochemical recurrence-free survival in patients with prostate cancer (multivariate Cox analysis; dichotomization using median)

| Gene | Name | Function | Publications for cancer* | Publications for prostate cancer* |
|---|---|---|---|---|
| **AKAP8** | A-Kinase Anchoring Protein 8 | Recruitment of PKA and other signaling molecules, chromosome condensation during mitosis | 11 | 0 |
| **ARMC9** | Armadillo Repeat Containing 9 | basal body protein encoded | 0 | 0 |
| **ATP6V1D** | ATPase H+ Transporting V1 Subunit D | component of vacuolar ATPase, associated with melanomas | 1 | 0 |
| **BLK** | B Lymphoid Tyrosine Kinase | B-cell receptor signaling and development | 96 | 6 |
| **C17orf67** | Chromosome 17 Open Reading Frame 67 | Uncharacterized | 0 | 0 |
| **C18orf32** | Chromosome 18 Open Reading Frame 32 | Activation of NF-kappa-B signaling pathway | 1 | 0 |
| **C22orf32** | Chromosome 22 Open Reading Frame 32 | regulatory subunit of the mitochondrial calcium uniporter complex (uniplex) | 1 | 0 |
| **CBR4** | Carbonyl Reductase 4 | mitochondrial fatty acid biosynthesis | 3 | 0 |
| **CCDC18** | Coiled-Coil Domain Containing 18 | nucleotide binding | 1 | 0 |
| **CHST7** | Carbohydrate Sulfotransferase 7 | generate sulfated glycosaminoglycan (GAG) during chondroitin sulfate biosynthesis, transfer sulfate to N-acetylgalactosamine | 6 | 0 |
| **CSTF3** | Cleavage Stimulation Factor Subunit 3 | polyadenylation and 3' end cleavage of pre-mRNAs | 2 | 0 |
| **CYSLTR1** | Cysteinyl Leukotriene Receptor 1 | receptor for cysteinyl leukotrienes mediating bronchoconstriction | 9 | 0 |
| **DBH** | Dopamine Beta-Hydroxylase | Catalyzation of the conversion of dopamine to norepinephrine | 41 | 0 |
| **DGAT2** | Diacylglycerol O-Acyltransferase 2 | Catalyzation of covalent binding of diacylglycerol to long chain fatty acyl-CoAs | 13 | 1 |
| **DKK2** | Dickkopf WNT Signaling Pathway Inhibitor 2 | embryonic development, agonist or antagonist of Wnt/beta-catenin signaling | 65 | 3 |
| **DLK1** | Delta Like Non-Canonical Notch Ligand 1 | regulator of cell growth, differentiation of several cell types including adipocytes | 174 | 11 |
| **DNM3** | Dynamin 3 | guanosine triphosphate (GTP)-binding protein associated with microtubules and vesicular transport | 12 | 0 |

| | | | | |
|---|---|---|---|---|
| **E2F3** | E2F Transcription Factor 3 | Regulation of the expression of cell-cycle genes | 298 | 24 |
| **EMR4P** | Adhesion G Protein-Coupled Receptor E4, Pseudogene | leukocyte adhesion and migration | 0 | 0 |
| **EPO** | Erythropoietin | promotes red blood cell production or erythropoiesis | 1087 | 19 |
| **ERI1** | Exoribonuclease 1 | histone mRNA decay after replication | 2 | 0 |
| **FAM170A** | Family With Sequence Similarity 170 Member A | Regulation of the expression of heat shock genes | 0 | 0 |
| **FZD2** | Frizzled Class Receptor 2 | Receptor for Wnt proteins,<br>coupled to the beta-catenin canonical signaling pathway | 45 | 1 |
| **GBAS** | Glioblastoma amplified sequence | vesicular transport, oxidative phosphorylation | 6 | 0 |
| **KIAA0196** | WASH Complex Subunit Strumpellin | inhibiting WASH nucleation-promoting factor (NPF) activity<br>recruitment and activation of the Arp2/3 complex | 5 | 4 |
| **KIF19** | Kinesin Family Member 19 | Response to elevated platelet cytosolic Ca2+ and Golgi-to-ER retrograde transport | 1 | 0 |
| **KLHL38** | Kelch Like Family Member 38 | Contributes to ubiquitin-protein transferase activity | 0 | 0 |
| **LAIR1** | Leukocyte Associated Immunoglobulin Like Receptor 1 | anchor for tyrosine phosphatase SHP-1,<br>may induce cell death in myeloid leukemias | 4 | 0 |
| **LILRA1** | Leukocyte Immunoglobulin Like Receptor A1 | regulation of immune responses | 1 | 0 |
| **LILRA3** | Leukocyte Immunoglobulin Like Receptor A3 | soluble receptor for class I major histocompatibility complex (MHC) antigens | 4 | 2 |
| **MAPK15** | Mitogen-Activated Protein Kinase 15 | transferase activity,<br>transferring phosphorus-containing groups,<br>protein tyrosine kinase activity | 10 | 0 |
| **NBEAL2** | Neurobeachin Like 2 | role in megakaryocyte alpha-granule biogenesis | 6 | 0 |
| **NCKIPSD** | NCK Interacting Protein With SH3 Domain | signal transduction,<br>function in the maintenance of sarcomeres and in the assembly of myofibrils into sarcomeres | 5 | 0 |
| **P2RY12** | Purinergic Receptor P2Y12 | platelet aggregation,<br>target for the treatment of thromboembolisms and other clotting disorders | 20 | 0 |
| **PABPC1P2** | Poly(A) Binding Protein Cytoplasmic 1 Pseudogene 2 | pseudogene, uncharacterized | 0 | 0 |
| **PCDHGA9** | Protocadherin Gamma Subfamily A 9 | establishment and function of specific cell-cell connections in the brain | 2 | 0 |
| **PDGFA** | Platelet Derived Growth Factor Subunit A | binding and activation of PDGF receptor tyrosine kinases,<br>developmental processes | 88 | 5 |
| **PDZK1** | PDZ Domain Containing 1 | regulation of the HDL receptor (cholesterol metabolism) | 36 | 0 |

| | | | | |
|---|---|---|---|---|
| **PIWIL4** | Piwi Like RNA-Mediated Gene Silencing 4 | development and maintenance of germline stem cells | 16 | 0 |
| **PPIL2** | Peptidylprolyl Isomerase Like 2 | protein folding, immunosuppression by cyclosporin A and infection of HIV-1 virions | 1 | 0 |
| **PSORS1C3** | Psoriasis Susceptibility 1 Candidate 3 (Non-Protein Coding) | RNA Gene, non-protein coding | 0 | 0 |
| **PXK** | PX Domain Containing Serine/Threonine Kinase Like | synaptic transmission,<br>ligand-induced internalization and degradation of epidermal growth factors | 5 | 1 |
| **RALB** | RAS Like Proto-Oncogene B | mediation of the transmembrane signaling initiated by certain cell surface receptors | 86 | 2 |
| **RFXAP** | Regulatory Factor X Associated Protein | central role in development and control of the immune system | 11 | 0 |
| **RMRP** | RNA Component Of Mitochondrial RNA Processing Endoribonuclease | cleavage of mitochondrial RNA at a priming site of mitochondrial DNA replication | 23 | 1 |
| **RPL18** | Ribosomal Protein L18 | component of the 60S ribosome subunit | 3 | 0 |
| **RPL27A** | Ribosomal Protein L27a | component of the 60S ribosome subunit | 8 | 0 |
| **SCP2** | Sterol Carrier Protein 2 | oxidation of branched chain fatty acids | 30 | 0 |
| **STC2** | Stanniocalcin 2 | regulation of renal and intestinal calcium and phosphate transport, cell metabolism, cellular calcium/phosphate homeostasis | 63 | 1 |
| **SYBU** | Syntabulin | activity-dependent presynaptic assembly during neuronal development | 0 | 0 |
| **TEKT3** | Tektin 3 | exact function of this gene is not known | 0 | 0 |
| **TFR2** | Transferrin Receptor 2 | mediates cellular uptake of transferrin-bound iron,<br>iron metabolism, hepatocyte function and erythrocyte differentiation | 39 | 1 |
| **TNKS** | Tankyrase | NAD+ ADP-ribosyltransferase activity | 126 | 1 |
| **UBE2L3** | Ubiquitin Conjugating Enzyme E2 L3 | ubiquitination of p53, c-Fos, and the NF-kB precursor p105 in vitro | 34 | 1 |
| **WRAP53** | WD Repeat Containing Antisense To TP53 | telomere synthesis | 30 | 0 |
| **ZCCHC5** | Zinc Finger CCHC-Type Containing 5 | transcriptional regulation, postnatal myogenesis, regulation of satellite cells self-renewal | 1 | 0 |
| **ZNF414** | Zinc Finger Protein 414 | transcriptional regulation | 1 | 0 |

*At manuscript submission date.
